# Supplementary material for: Seminoma and Embryonal Carcinoma Footprints Identified by Analysis of Integrated Genome-Wide Epigenetic and Expression Profiles of Germ Cell Cancer Cell Lines
Source: PLoS One. 2014 Jun 2;9(6):e98330. doi: 10.1371/journal.pone.0098330 (PMC4041891; doi:10.1371/journal.pone.0098330)
Supplement: File S1 — ZIP file containing DMRforPairs output for significant regions. Please start from the html files. (ZIP) [file pone.0098330.s008.zip › figures/28993.pdf]

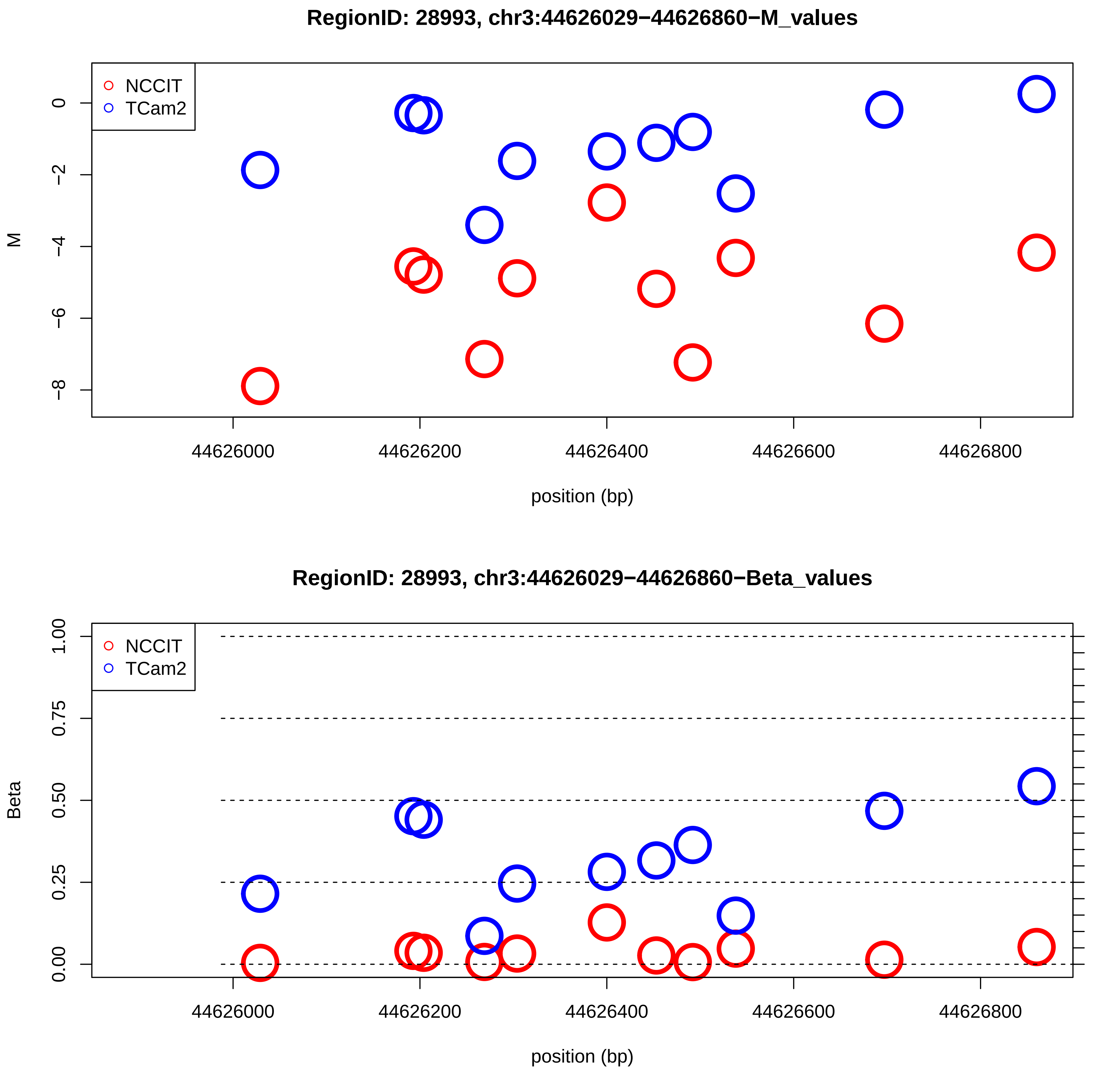

RegionID: 28993, chr3:44626029−44626860−M_values
NCCIT
TCam2
M
0
−2
−4
−6
−8
44626000
44626200
44626400
44626600
44626800
position (bp)
RegionID: 28993, chr3:44626029−44626860−Beta_values
NCCIT
TCam2
Beta
1.00
0.75
0.50
0.25
0.00
44626000
44626200
44626400
44626600
44626800
position (bp)

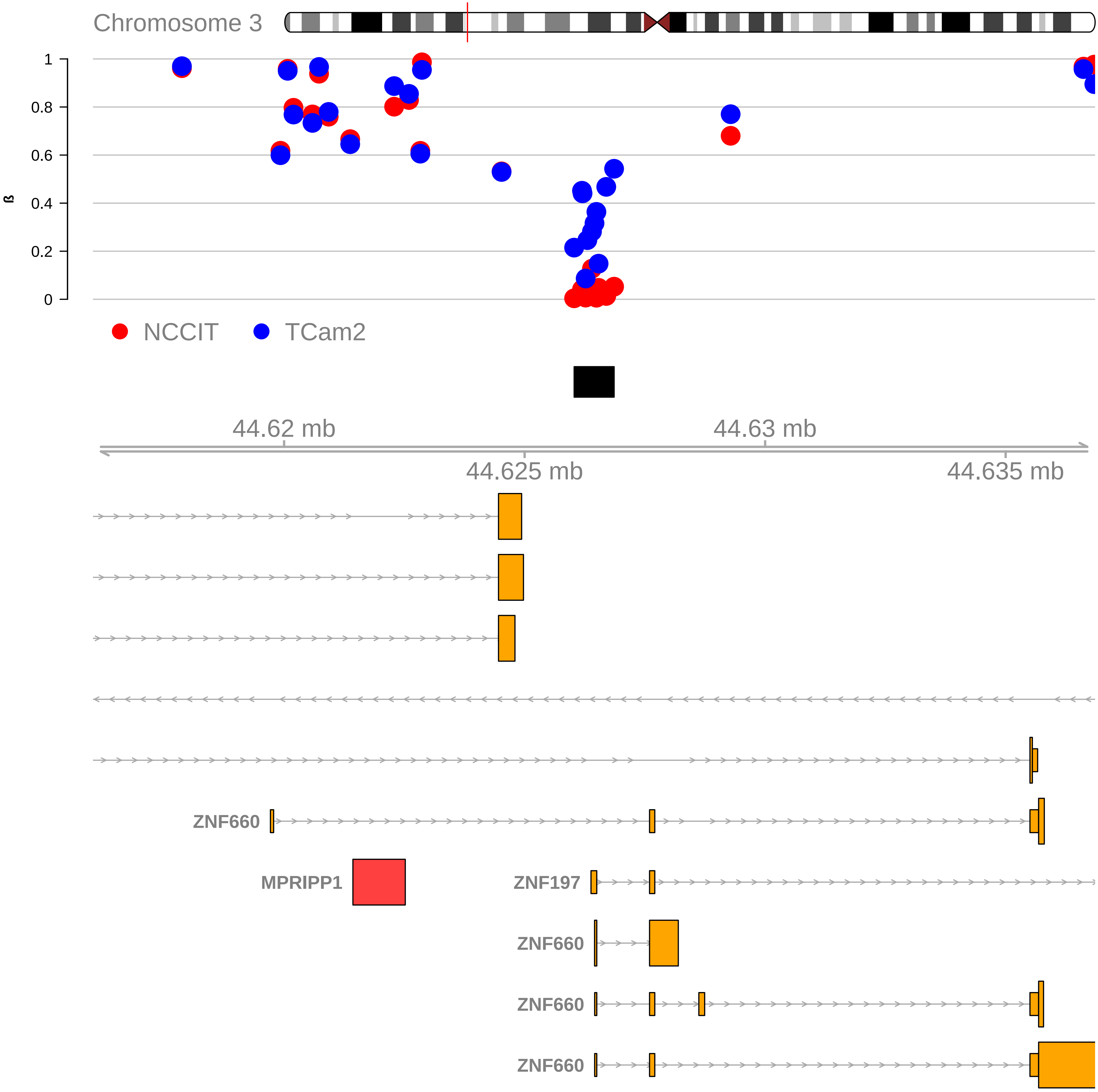
Chromosome 3
1
0.8
0.6
0.4
0.2
0
β
NCCIT
TCam2
44.62 mb
44.63 mb
44.625 mb
44.635 mb
ZNF660
MPRIPP1
ZNF197
ZNF660
ZNF660
ZNF660
